# Memoryless chemotaxis with discrete cues

**DOI:** 10.1098/rsif.2024.0100

**Published:** 2024-07-31

**Authors:** Jacob Knight, Paula García-Galindo, Johannes Pausch, Gunnar Pruessner

**Affiliations:** ^1^ Department of Mathematics, Imperial College London, South Kensington, London SW7 2BZ, UK; ^2^ Department of Chemical Engineering and Biotechnology, University of Cambridge, Philippa Fawcett Drive, Cambridge CB3 0AS, UK

**Keywords:** chemotaxis, search strategy, cell navigation, mathematical biology

## Abstract

Biological systems such as axonal growth cones perform chemotaxis at micrometre-level length scales, where chemotactic molecules are sparse. Such systems lie outside the range of validity of existing models, which assume smoothly varying chemical gradients. We investigate the effect of introducing *discrete* chemoattractant molecules by constructing a minimal dynamical model consisting of a chemotactic cell without internal memory. Significant differences are found in the behaviour of the cell as the chemical gradient is changed from smoothly varying to discrete, including the emergence of a homing radius beyond which chemotaxis is not reliably performed.

## 1. Introduction

To sense its environment, a cell is limited to measuring the occupation of the receptors distributed over its surface [1]. Using this measurement, the cell can detect diffusive particles, which allow it to perform chemotaxis: navigation in an environment with cue chemicals that may attract or repel the cell [2]. Chemotaxis is an important biological function in single cells since it allows the movement towards a more favourable region for survival and growth [3]. In multi-cellular organisms, it ensures that cells are in the right place at the right time, which is essential for basic processes such as wound healing [3]. Additionally, unwanted or unregulated chemotaxis can become a contributing factor in diseases such as cancer, asthma or arthritis [3].

A cell’s ability to sense gradients from the stochastic arrival of ligands on its receptors has an impact on how efficiently it performs chemotaxis. The physical limits of this process were first calculated for concentration sensing [1] and later for gradient sensing [4,5]. How the measurement of a gradient is performed differs between cell types. In eukaryotic cells, the measurement is commonly done directly, where a single cell measures the gradient across the diameter of its body [6]. Eukaryotic cells, such as slime mould *Dictyostelium discoideum* (Dicty) [7], yeast *Saccharomyces cerevisiae* [8] and immune cells like neutrophils and leukocytes [9], are remarkably capable of detecting small changes in particle flux, often operating close to physical limits.

Chemotaxis can also be performed by cell parts such as the growth cone of neurons, where it is called axon guidance [10]. Axons have been shown to successfully perform chemotaxis in regions where the average difference in the number of molecules between the high- and low-concentration sides of a growth cone is just *one-fifth of a molecule* [11]. Existing calculations of physical limits on chemotactic performance represent the density of chemoattractant particles as a smoothly varying quantity. This contrasts with the reality that chemotaxis can occur on length scales where the size of the chemotactic agent (i.e. cell or cell part) is of a similar order to the distance between molecules of chemoattractant. This raises the following question: how does the reality that chemoattractants comprise *discrete* molecules, rather than a smoothly varying field, affect chemotaxis?

We address this question by analytically and numerically characterizing an idealized model consisting of a spherical cell in the presence of a point source of chemoattractant in three dimensions. Our model cell has perfect single-molecule sensitivity (each ligand is recorded), moves at constant speed and instantaneously reorients in the direction of the most recently absorbed ligand. The latter property is reminiscent of the greedy algorithm in which the locally optimal solution is followed [12]. As the greedy algorithm results in the best possible response towards finding the source in the absence of information processing and memory, we consider our model as an optimal chemotaxis strategy for a memoryless cell exposed to discrete cue particles.

This article is organized in the following way. We first define a model cell inspired by Endres and Wingreen’s perfectly absorbing sphere [5] that moves using the greedy algorithm, as well as the surrounding environment of diffusive particles. Next, we analytically derive an effective cell velocity, which represents an ensemble average of the greedy cell dynamics. We find a threshold distance beyond which an ensemble of cells will move away from the source on average, which we term the *homing radius*. We validate our model by comparing the dynamics of numerical simulations with analytic predictions in different environment regimes. Finally, we highlight examples of biological relevance as well as limitations of the model and suggest directions for future research.

## 2. Methods

In this section, we introduce and analytically characterize our model of a chemotactic cell in three dimensions, whose motion is governed by the greedy algorithm.

### 2.1. Model

**Figure 1.**
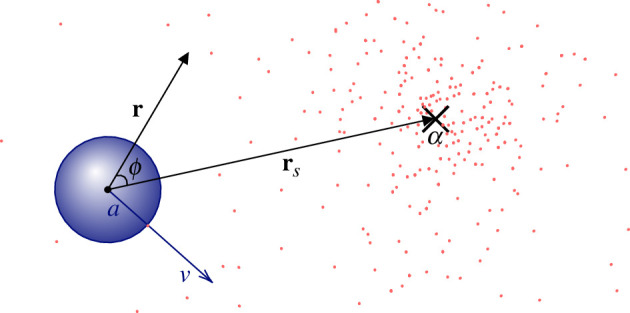
Schematic of the model. The centre of the cell (dark blue) is the origin of the coordinate system. Point-like cue particles (red dots) are released with Poissonian rate α from a point source located at $r_{s}$ and diffuse with diffusion constant D. The cell of radius a absorbs cue molecules on contact and moves with constant speed v in the direction of incidence of the last cue molecule, as illustrated. The vector r denotes a position where the density of cue particles is evaluated. The angle ϕ∈[0,π] denotes the polar angle between r and $r_{s}$.

Consider a spherical cell of radius a in the presence of a point source that releases point-like cue particles with Poissonian rate α>0. Cue particles represent molecules of a generic chemoattractant and diffuse with diffusivity D. The cell moves with constant speed v and does not experience rotational or translational diffusion. The cell can detect cue particles when they collide with its surface. Cue particles are absorbed upon contact with the cell surface, as in the *perfectly absorbing sphere* model considered in [5]. The cell has no memory, i.e. no capacity to store information. The process terminates successfully if the cell reaches the source. This set-up is illustrated in figure 1. We study the model in three dimensions rather than two because here, even without a source for guidance, a randomly moving cell would eventually find any specific point.

The motion of the cell in time is determined by the way it responds to incident cue molecules. Given that the cell has no memory, any action it takes must be local in time. In optimization problems, a greedy algorithm is one which always takes the best immediate, or local, solution [12]. The optimal generic strategy for the memoryless cell must therefore be to adopt a greedy algorithm that maximizes the probability of reaching the source of diffusive particles. Without the benefit of memory, the optimal instantaneous response to receiving a cue particle is to move in the direction of incidence of the most recently absorbed cue particle.

**Figure 2.**
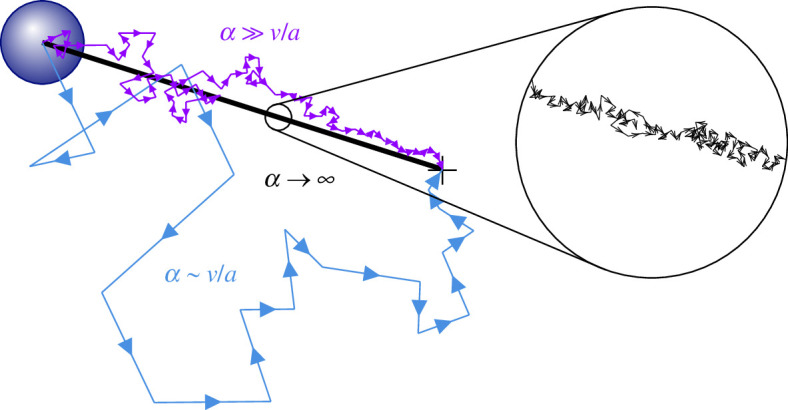
Schematic of trajectories at various cue particle release rates. Runs between collisions with cue particles are longer at a low release rate α since the density of cue particles is lower. As the release rate tends to infinity (α→∞), runs become infinitely short and the cell trajectory tends towards a straight line. Labels compare the magnitude of the release rate α to the time taken for the cell to move by a single radius, a/v. The ratio of these time scales ε=αa/v characterizes the cell trajectory, as derived in §2.3.

The motion of the cell is deterministic in the limit α→∞ since the probability distribution of cue arrivals on the cell surface is exhaustively sampled and the time between collisions with cues vanishes. Conversely, for finite α>0, the cell will undertake a random walk whose properties are determined by the release rate α of the cue particles, and the velocity v and radius a of the cell. These trajectories are illustrated schematically in figure 2. We subsequently construct an effective velocity $v_{\text{eff}}$, which describes the speed with which the cells approach the source.

The cue particle density ρ(r) at position r owing to a source with release rate α at $r_{s}$ is given as the solution to Poisson’s equation


(2.1) $$-D\nabla^{2}\rho (r)=\alpha \delta (r_{s}-r)\;,$$

under the boundary conditions


(2.2a) ρ(|r|=a)=0,

(2.2b) $$\underset{|r|\to \infty }{\lim}\rho (r)=0.$$

Boundary condition (2.2a) enforces the complete absorption of cue particles on the surface of the cell, whereas boundary condition (2.2b) allows cue particles to increasingly dilute away from the source.

The solution to equation (2.1), which satisfies the boundary conditions (2.2a) and (2.2b), is identical (in the region |r|>a) to the solution of


(2.3a) $$-D\nabla^{2}\rho (r)=\alpha \delta (r_{s}-r)+\alpha^{\prime }\delta (r_{s}^{\prime }-r),$$

(2.3b) $$\underset{|r|\to \infty }{\lim}\rho (r)=0.$$

The boundary condition (2.2a) on the diffusion equation (2.1) has been implemented by introducing a so-called ‘image charge’, whose strength $\alpha^{\prime }=-\alpha \cdot a/r_{s}$ and position $r_{s}^{\prime }=a^{2}/r_{s}^{2}\cdot r_{s}$ are chosen such that (2.2a) is satisfied [13]. The solution is


(2.4) $$\begin{array}{c} \rho \left(r\right)=\frac{\alpha }{4\pi D}\left[\frac{1}{{\left(r^{2}+r_{s}^{2}-2r\cdot r_{s}\right)}^{\frac{1}{2}}}-\frac{1}{{\left(r^{2}r_{s}^{2}/a^{2}+a^{2}-2r\cdot r_{s}\right)}^{\frac{1}{2}}}\right]. \end{array}$$

The flux into the cell is proportional to the gradient of the density evaluated at the cell surface and depends only on the polar angle ϕ∈[0,π] of the considered point r on the cell surface with respect to the direction of the source ${\hat{r}}_{s}$,


(2.5) $$J(\phi )=-D\partial_{r}\rho \left(r\right){|}_{r=a}\;\;\;=\frac{\alpha }{4\pi a}\cdot \frac{r_{s}^{2}-a^{2}}{{\left(r_{s}^{2}+a^{2}-2ar_{s}cos\phi \right)}^{\frac{3}{2}}},$$

valid for $r_{s}>a$ such that the source resides outside the cell and independent of the diffusivity D, as discussed further in §3.1. The rate of cue molecule arrivals through the area of the cell surface between angles in the range [ϕ,ϕ+dϕ] with respect to the source is given by J(ϕ)dA(ϕ), where $\text{d}A\left(\phi \right)=2\pi a^{2}sin\left(\phi \right)\text{d}\phi$ is the area of the cell surface with a polar angle in the range [ϕ,ϕ+dϕ]. Once appropriately normalized by the total rate of arrivals of cues at the cell surface, $\int \text{d}\phi J(\phi )A(\phi )=\alpha a/r_{s}$, this gives a probability density for the angle of arrival of cue molecules on the cell surface,


(2.6) $$p\left(\phi \right)=\frac{r_{s}}{2}\frac{\left(r_{s}^{2}-a^{2}\right)sin\left(\phi \right)}{{\left(r_{s}^{2}+a^{2}-2ar_{s}cos\phi \right)}^{\frac{3}{2}}},$$

with $r_{s}>a$

### 2.2. Effective velocity at infinite release rate

In the limit α→∞, the distribution of cue particles tends towards a smoothly varying field. Since the total rate of arrivals into the cell $\alpha a/r_{s}$ also goes to infinity, the time between cue particle arrivals vanishes, and the zig-zag motion of the cell becomes a straight line as illustrated in figure 2. Its effective velocity towards the source, $v_{\text{eff},\infty }(r_{s})=-\text{d}r_{s}/\text{d}t$, is the projection of the cell motion in the direction of the source,


(2.7) $$\begin{array}{c} v_{\text{eff},\infty }\left(r_{s}\right)=E\left[vcos\phi \right]=v\int_{0}^{\pi }cos\left(\phi \right)p\left(\phi \right)\text{d}\phi ,\\ =\frac{av}{r_{s}}. \end{array}$$

Since this describes the deterministic motion of the cell, $v_{\text{eff},\infty }(r_{s})$ can be integrated to obtain the long-time cell trajectory


(2.8) $$r_{s}(t)=\sqrt{r_{0}^{2}-2avt}\;,$$

where $r_{0}(\geq a)$ is the initial distance between the source and the centre of the cell.

### 2.3. Effective velocity at finite release rate

When cue particles are released at a finite rate α, there is a random, finite time between cue arrivals on the cell surface. As such, this executes a random walk, as illustrated in figure 2. In the following, we develop the notion of the resulting effective $v_{\text{eff},\alpha }(r_{s})$. We consider a cell located at distance $r_{s}$ from the source, which receives a cue particle at a polar angle ϕ relative to the source. The random variable describing the time before the next collision with a cue particle is denoted Δt. Each of these journeys along a straight line is referred to as a ‘run’ in the following. The change in radial distance of the cell to the source during a single run is denoted by $\Delta r_{s}$,


(2.9) $$\Delta r_{s}\left(r_{s},\phi ,\Delta t\right)=\sqrt{r_{s}^{2}+v^{2}\Delta t^{2}-2r_{s}v\Delta tcos\left(\phi \right)}-r_{s}\;.$$

The effective velocity for a finite release rate of cue particles can be related to the chemotactic index [5,14] of a single run. The chemotactic index is defined as the distance moved in the direction of the source divided by the total distance moved by the cell [5],


(2.10) $$\text{CI}=-\frac{\bar{\Delta r_{s}}}{v\cdot \bar{\Delta t}}\;,$$

where the expectation values $\bar{•}$ are taken with respect to the angular distribution of cue arrivals on the cell surface and the probability distribution of run durations,


(2.11) $$\bar{\Delta r_{s}}\left(r_{s}\right)=\int_{0}^{\pi }\text{d}\phi \int_{0}^{\infty }\text{d}\Delta t\;\Delta r_{s}\left(r_{s},\phi ,\Delta t\right)\;p\left(\phi ,\Delta t\right)\;,$$

where p(ϕ,Δt) denotes the joint probability of a cue at polar angle ϕ resulting in a run of duration Δt. At finite release rate of cue particles (and thus finite incidence rate on the cell surface), the velocity of the cell towards the source is a random number in the range [−v,v]. We define its effective value $v_{\text{eff},\alpha }(r_{s})$ via the chemotactic index $v_{\text{eff},\alpha }\left(r_{s}\right)=-v\;\text{CI}=\bar{\Delta r}\left(r_{s}\right)/\bar{\Delta t}\left(r_{s}\right)$.

To evaluate $\bar{\Delta r}\left(r_{s}\right)$ and $\bar{\Delta t}\left(r_{s}\right)$, we express the joint probability density p(ϕ,Δt)=p(ϕ)p(Δt|ϕ) as the product of the angular distribution (2.6) and the distribution of the run duration Δt conditioned on a collision at an angle ϕ. Since the distribution of cue particles is inhomogeneous, cue-cell collisions are governed by a Poisson process with rate dependent on the position of the cell relative to the source. The rate of cue collisions with the cell $\lambda (r_{s})$ is equal to the total flux of cue particles into the cell, $\lambda (r_{s})=\int \text{d}\phi J(\phi )A(\phi )=\alpha a/r_{s}$. For a cell that experiences a collision occurring at a distance $r_{s}$ and polar angle ϕ relative to the source and continues to move for a further time t, the total rate of cue arrivals as a function of time becomes


(2.12) $$\lambda \left(t\right)=\frac{\alpha a}{\sqrt{r_{s}^{2}+v^{2}t^{2}-2r_{s}vtcos\left(\phi \right)}}\;.$$

The probability density of Δt conditioned on cue arrival at an angle ϕ is hence given by


(2.13) $$p\left(\Delta t|\phi \right)=\lambda \left(\Delta t\right)exp\left(-\int_{0}^{\Delta t}\text{d}t\lambda \left(t\right)\right),$$

which is calculated explicitly in electronic supplementary material, appendix I. With the probability densities known, the mean change in radial coordinate $\bar{\Delta r_{s}}$ and the mean run duration $\bar{\Delta t}$ can be expressed in terms of a dimensionless parameter $\varepsilon =\frac{\alpha a}{v}$ (electronic supplementary material, appendix I),


(2.14a) $$\bar{\Delta r_{s}}\left(r_{s}\right)=\frac{a\varepsilon -r_{s}}{1-\varepsilon^{2}}\;,$$

(2.14b) $$\bar{\Delta t}\left(r_{s}\right)=\frac{1}{v}\;\frac{a-\varepsilon r_{s}}{1-\varepsilon^{2}}\;.$$

These results are valid only for ε>1 as both $\bar{\Delta r}\left(r_{s}\right)$ and $\bar{\Delta t}\left(r_{s}\right)$ diverge for ε≤1. This condition has a physical significance, which is discussed in §3. From equation (2.10), the chemotactic index of the cell is


(2.15) $$\text{CI}\left(r_{s}\right)=\frac{a\varepsilon -r_{s}}{r_{s}\varepsilon -a}\;,$$

and the effective velocity is therefore


(2.16) $$v_{\text{eff},\alpha }\left(r_{s}\right)=v\;\frac{a\varepsilon -r_{s}}{r_{s}\varepsilon -a}\;.$$

The signs of $\bar{\Delta r}\left(r_{s}\right)$ and hence $\text{CI}(r_{s})$ and $v_{\text{eff},\alpha }(r_{s})$ change at a radius which we term the *homing radius*,


(2.17) $$r_{h}=a\varepsilon =\frac{\alpha a^{2}}{v}.$$

The mechanism by which this occurs is discussed in §3.

**Figure 3.**
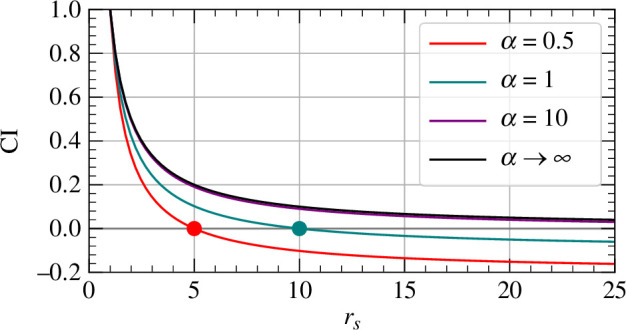
Plot of the chemotactic index $\text{CI}(r_{s})$ for various values of α, measured in units of $s^{-1}$. For finite values of α, there is a homing radius $r_{h}$ defined in equation (2.17) beyond which the chemotactic index, and hence the effective velocity, of the cell is negative, corresponding to a net migration of cells away from the source. The homing radii at α=0.5,1 are marked on the plot with solid points. The chemotactic index for finite α defined in equation (2.10) converges to that of infinite α as denoted in equation (2.7) (noting that $v_{\text{eff}}(r_{s})=-v\;\text{CI}(r_{s})$). For each curve the cell radius and velocity are a=1 and v=0.1, respectively.

Rather than the deterministic speed $v_{\text{eff},\infty }(r_{s})$ of a cell, $v_{\text{eff},\alpha }(r_{s})$ instead describes the rate of approach towards the source of the mean position of an ensemble of independent cells (each with an independent set of cue particles) over a single run. After one run, cells will be spread out in space with some nonlinear distribution. Since equation (2.16) applies only to an ensemble at a single point in space and is nonlinear in $r_{s}$, the subsequent motion of cells cannot be described by integrating equation (2.16) as it was in equation (2.8). Nonetheless, equation (2.7) is recovered in the limit α→∞


(2.18) $$\underset{\alpha \to \infty }{lim}v_{\text{eff},\alpha }\left(r_{s}\right)=\frac{av}{r_{s}}=v_{\text{eff},\infty }\left(r_{s}\right),$$

as illustrated in figure 3.

### 2.4. Comparison with simulation

We numerically simulated the system described in §2.1 in order to validate our calculations and to see how long the effective velocity provided a good description of average cell motion. An outer boundary was introduced at a large distance $R_{c}\gg a,r_{s},r_{0}$ as a cut-off at which a cell was deemed irretrievably lost on its way to the source. This outer boundary also absorbed cue particles, which was necessary for the simulation to reach a steady state. The deviation from the density distribution equation (2.4) that this caused was found to be negligibly small.

The system was initialized without cue particles, with the absorbing cell placed a distance $r_{s}=r_{0}$ from the source. Cue particles were subsequently released from the source at rate α and diffused with diffusion constant D. The system was allowed sufficient time to reach a steady state with the cell remaining stationary, i.e. not responding to cues. After this time and once the first cue arrived, the clock was reset to t=0 and the cell began to move according to the greedy algorithm (§2.1), and its position was recorded as a function of time. Each trial terminated when the cell reached the source or the outer boundary. Upon termination of the trial, the cell was reset to its initial position, without changing the positions of cue particles. Sufficient time was allowed for the system to return to its original steady-state distribution, with the cell stationary. This was repeated 1000 times in each simulation.

**Figure 4.**
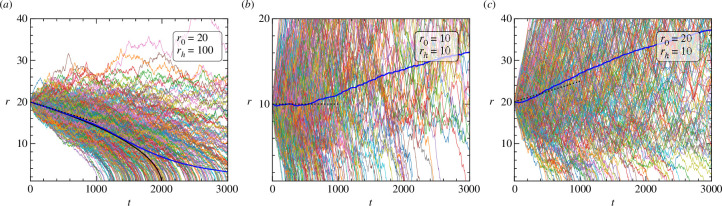
Sample trajectories of ensembles of cells initialized at $r_{0}<r_{h}$ (*a*), $r_{0}=r_{h}$ (*b*) and $r_{0}>r_{h}$ (*c*). Multi-coloured lines represent the trajectories of individual cells, while the blue solid line denotes the mean distance of the cells from the source averaged over 1000 trajectories. The black solid line in panel (*a*) shows the trajectory described by equation (2.8) for infinite release rate and the black dotted lines in all panels show the predicted instantaneous velocity from equation (2.16). The initial motion of the centre of mass of the cells is described well by equation (2.8). Qualitatively, the observed long-time positive deviation from theoretical predictions occurs because some proportion of the initial ensemble runs away from the source towards large r. This part of the ensemble dominates the mean particle position in the long-time regime since they reach increasingly large r. For all runs, v=0.1 and a=1. The other parameters are (*a*) $r_{0}=20$, α=10, (*b*) $r_{0}=10$, α=1, (*c*) $r_{0}=20$, α=1.

Sample trajectories $\left\{r_{s}(t)\right\}$ from this simulation are shown in figure 4. The simulations confirm that the effective velocity defined in equation (2.16) successfully predicts the initial velocity of the ensemble-averaged position of the cells. It also demonstrates that equation (2.8) provides a good approximation of the long-term trajectory of the centre of mass of an ensemble of particles with a large but finite release rate.

Deviations of the numerics from the predicted behaviour have two causes. First, we consider the motion of an ensemble of cells all starting at a single point, not accounting for their subsequent spread in space according to the independent collision events for each cell. Second, our theory does not account for correlations in the location of the cue particles owing to the ‘wake’ left behind by the cell. These two points are further discussed in the following section.

## 3. Discussion

### 3.1. Chemotaxis in arbitrarily shallow gradients

Remarkable sensitivity to chemical gradients is a feature of eukaryotic cells as well as smaller systems like axon growth cones. Eukaryotic cells can sense gradients as shallow as 1–5% across their body length [7]. Understanding the mechanism by which this is achieved is a long-standing open question in the study of chemotaxis. As detailed in the following, in our model the chemical gradient in which the cell navigates can be made arbitrarily shallow without affecting chemotactic performance.

From equation (2.4), the local chemical gradient at a cell’s surface is proportional to the chemoattractant release rate divided by the diffusivity, $\nabla_{r}\rho (r){|}_{r=x}\propto \alpha /D$. However, equation (2.5) shows that the flux into the cell is proportional to the release rate but independent of the diffusivity, J(x)∝α. As such, the chemical gradient in the vicinity of a cell can be made arbitrarily small by increasing the diffusivity D without affecting the flux incident on the cell and hence its ability to perform chemotaxis. Intuitively, increasing D will spread out the chemoattractant profile, thereby decreasing the local density around the cell. This is compensated for by the higher mean-square displacement of nearby cue particles, which enter the cell more frequently.

Existing literature posits that cells can sense gradients either by measuring the chemical *flux* into their surface or the chemical density inside their volume [5]. We argue that the diffusion independence of particle flux into the cell surface provides a physical explanation for the ability of flux-sensing cells to navigate in extremely shallow gradients. This is not only true in our model of a spherical cell; we demonstrate in electronic supplementary material, appendix C that in three dimensions molecular flux into a cell is independent of diffusivity *for cells of any shape* and for *any arrangement of sources or sinks of chemoattractant* under the boundary condition $\underset{|x|\to \infty }{\lim}\rho (x)=0$, providing a convincing explanation for the ability of cells to navigate in shallow chemical gradients.

### 3.2. Sign change in effective velocity

**Figure 5.**
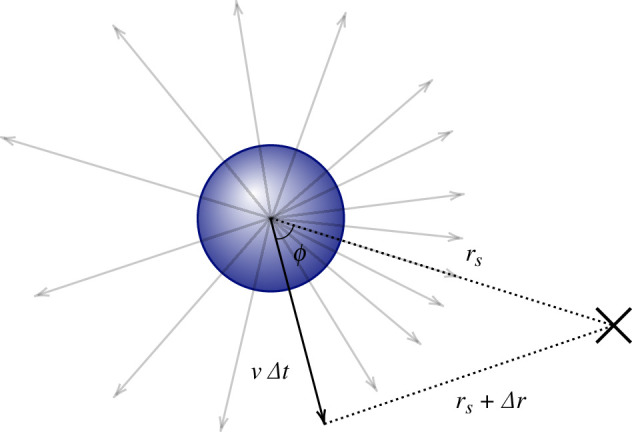
Schematic illustration of the variation of cell runs with ϕ at finite α. The length of grey arrows represents the mean run length $\bar{\Delta r\left(\phi \right)}$, while the density of grey arrows represents the distribution of arrivals on the cell surface. Although the angular distribution of runs is biased towards the source, runs away from the source tend to be longer (illustrated as longer grey arrows) since the density of cue particles is lower further from the source. Beyond the homing radius $r_{h}$, this leads to a negative chemotactic index and hence a net migration of cells away from the source.

When α is finite, there is a homing radius defined in equation (2.17) beyond which the chemotactic index and hence the effective velocity are negative. Two competing factors affect the effective velocity of the cell: the bias of the angular distribution (equation (2.6)) towards the source, and the fact that runs away from the source tend to be longer. The bias of the angular distribution is visible by rescaling p(ϕ) in equation (2.6) to be the density per solid angle, p(ϕ)/sin(ϕ), which has its maximum at ϕ=0 and its minimum at ϕ=π. The competing effect is that the position-dependent cue collision rate λ(r)∝1/r decreases with distance from the source. As a result, runs away from the source persist for longer. The cell is less likely to move away from the source than towards it, but if it does, it takes longer for a cue particle to collide with it and change its direction. This effect is illustrated schematically in figure 5.

At the homing radius, these effects cancel out, leading to no net movement of the cell on average. Of an ensemble of cells initialized at $r_{s}=r_{h}$ and allowed to complete their trajectories, more than half would move in the direction of the source over their first run. This is because the bias in angular distribution of cue arrivals means that more than half of the cells would take an initial step towards the source, into a region where $v_{\text{eff},\alpha }>0$. To calculate the proportion of cells that eventually succeed in arriving at the source is challenging because it involves correlated sequences of runs. An analysis to be explored in future work is outlined in electronic supplementary material, appendix II.

The homing radius is derived solely from microscopic system parameters. This stands in contrast to previous approaches, which require the introduction of an external cut-off on the signal-to-noise ratio of the arrivals on the cell surface [15]. Our model also makes a concrete prediction about the effective velocity of the cell.

Further intuition is provided by the observation from equation (2.14b) that the homing radius is the distance from the source at which the mean run length of the cell is equal to the cell radius, $v\bar{\Delta t}\left(r_{h}\right)=a$. This demonstrates that the length scale at which the molecular description of chemoattractant differs significantly from the smoothly varying description is that at which the distance between interactions with cues is of the order of the cell radius.

### 3.3. Range of validity of results

The condition ε>1 which is required to obtain equations (2.14a)–(2.16) has a number of physical interpretations. Multiplying both sides of the inequality by a yields $a\varepsilon =r_{h}>a$, i.e. the cell radius must be strictly less than the homing radius of the system. If this condition was broken, cells could be initialized at $r_{s}=a\geq r_{h}$, and would instantly be in contact with the source despite being outside the homing radius, which should imply that they move away from the source on average. Even if the cell touches the source, it would move away on average since $a>r_{h}$.

The parameter ε can be expressed as the ratio of two time scales, $\varepsilon =\alpha a/v=\tau_{b}/\tau_{r}$, where $\tau_{b}=a/v$ is the time taken for the cell to move a distance equal to its radius and $\tau_{r}=1/\alpha$ is the mean time between the release of cue particles. Requiring ε>1 therefore corresponds to demanding a sufficiently high cue release rate that the cell does not travel more than one radius in the mean time between releases.

Intuition can be gained from the fact that the probability distribution of run durations is governed by a power law: as Δt→∞, $p\left(\Delta t\right)\propto \Delta t^{-1-\varepsilon }$. It is evident that the integral $\bar{\Delta t}=\int_{0}^{\infty }\text{d}\Delta t\Delta tp\left(\Delta t\right)$ converges only for ε>1. Similar reasoning applies to $\bar{\Delta r}$. In qualitative terms, if the cell runs at an angle ϕ=π, and ε≤1, the expected time to encounter a cue particle diverges, so that expected step duration and length diverge as well.

### 3.4. Model assumptions

Several assumptions made in this model are stated in §2.1. One assumption is that the cell is not affected by translational or rotational diffusion. We expect that cell diffusion can be superimposed and would decrease the chemotactic index, corresponding to impaired cell performance. This could be implemented by replacing the denominator of equation (2.12) with the expected radial coordinate of an appropriately initialized active Brownian particle. Although mathematically feasible, this lies beyond the scope of the current work. Furthermore, values of initial distances, velocities and diffusion constants realistic to eukaryotic cells produce Péclet numbers in the thousands, meaning that corrections are expected to be small.

The model also assumes that the cell reorientation occurs instantaneously. In biological systems, rearrangement of cellular motility apparatus takes a non-negligible amount of time [16]. We argue that neglecting cell reorientation time is justified in each of two limits in our model. In the limit of sparse cue particles and hence few tumbles, the time taken for reorientation will be small compared with the time spent in motion. In the limit of dense cue particles (α→∞), the flux into the cell becomes infinite and trajectory of the cell becomes a straight line (see figure 2). The cell would therefore remain stationary if finite time was required for the cell to reorientate. In this limit, the cell moves according to an effective average over arrivals on its surface (despite performing no computation itself) and can be considered to be self-propelling in a single deterministic direction with velocity given by equation (2.7). Corrections between these limits could be introduced in extensions to the model.

The distribution of cue particles was assumed to be quasistatic, such that the density profile of cue particles in the system is always given by the solution to equations (2.3a) and (2.3b). This is valid when the time scale for a cue particle to diffuse some distance x is much smaller than the time for the cell to self-propel the same distance, equivalent to the condition D/(av)≫1. When this condition is not satisfied, the cell leaves a low-density region, or ‘shadow’, in its wake [17]. Capturing these correlations is very difficult, but neglecting them is justified since cue particles are far smaller than the cell, with diffusivity D orders of magnitude greater than that of the cell (and greater than the product av, as discussed above). Simulation parameters to generate figure 4 were chosen such that D/av=10, and it was checked that varying D did not significantly affect the statistics of observed trajectories.

This model also assumes that cue particles have an infinite lifetime after release. Recent work has shown that the generation of morphogen gradients in early *Drosophila* embryos and eggs is consistent with the synthesis-diffusion-degradation (SDD) model [18,19]. This corresponds to equation (2.1) with an additional term to represent the decay of cue particles. Although this equation cannot be solved analytically with the boundary conditions given in equations (2.2a) and (2.2b), a numerical analysis following the same steps would be tractable and an interesting avenue for further investigation.

The cell in the present model responds to *every* cue arrival on its surface, in contrast to real cells which have imperfect reception of ligands arriving on their surface. This can be accounted for by rescaling α by the proportion of arriving particles that will be absorbed; if only 1% of particles arriving on the surface produce a response from the cell, then α should be rescaled by the corresponding factor of 1/100.

A limitation of the present theory was that an effective velocity could only be calculated for an ensemble of cells initialized at the *same* distance from the source. After a short time, such an ensemble will be spread out in space, each cell moving according to the first arriving cue. This makes the subsequent calculation of an effective velocity of the centre of mass of the cells difficult, since the effective velocity applies only to an ensemble at the same distance from the source. As a result, we can predict exactly only the long-time trajectories of cells in the limit of α→∞, where the trajectories are deterministic. For finite α, the effective velocity describes the motion of the mean distance of the cells from the source only for a short time, approximately Δt.

### 3.5. Biological context

In biological systems, cells establish a sense of distance and direction through chemical cue gradients [20,21]. Most existing models, including widely used works [2,22], treat the concentration of chemoattractant as a continuously varying field. This commonly used mean-field approach to estimate concentration and gradient neglects the particle nature of chemoattractants. However, in the low-density regime, the discreteness of cues affects the qualitative behaviour of cells. It is well established that eukaryotic cells can adopt a favourable direction by considering only a few binding events of chemical cues [10,11,23]. Even at high chemoattractant densities, eukaryotic cells adopt their direction on the time scale of the first binding events [24]. Indeed, a simple model has recently shown how the first bindings can confer enough information to accurately estimate the source direction at a close enough distance [25].

In the present model, we analyse the impact of the particle nature of chemoattractants through a dynamical and perfectly sensitive cell that has no intricate processing or memory. The cell optimally searches the source using local information, an approach to understanding chemotaxis similar in spirit to infotaxis [26]. However, the infotaxis strategy considers the exploitation versus exploration trade-off, while the present greedy strategy takes an exploitation approach only as it moves instantaneously after the first hit, with no recall of previous hits. A potential future direction for the greedy cell model involves the integration of memory, with the expectation that the homing radius will expand or disappear. Recent work by Brumley *et al*. [27] provides a useful theoretical and experimental comparison with which more detailed future versions of the model can be compared.

Although the model presented in this work is not intended to replicate the spatial gradient sensing done by real cells, it more closely resembles the spatial gradient sensing usually performed by eukaryotic cells than the temporal gradient sensing of bacteria. This model expands on previous analysis of the *temporal* gradient sensing with discrete cues executed by a perfectly absorbing sphere [28]. Bacteria typically execute run-and-tumble dynamics, during which gradient sensing occurs over an extended period of time. However, the model cell in this chapter uses local information to direct its movement instantaneously, which relates to eukaryotic direct spatial gradient measurement, where the cell estimates the gradient across the diameter of its body [29,30]. Theory has been developed to compare the effectiveness of spatial and temporal gradient sensing, which accounts for the discrete nature of cue particles by modelling their arrivals as Poisson-distributed discrete events, without explicitly modelling discrete cues [31].

The eukaryotic Dicty cell is a particularly well-studied model organism for chemotaxis. It has a typical velocity of one to two body lengths per minute, placing them well within the regime D/av≫1 at room temperature. An earlier investigation by Endres and Wingreen [5] provided strong evidence that Dicty cells sense gradients over their surface and move accordingly. The model presented here extends their results to a regime where concentration is low enough that cue particles are sparse.

An example of such a system is the chemotaxis of immune cells such as macrophages or neutrophils towards a wound infection site [32]. We use our model to provide a ballpark estimate of the maximum desirable speed of an immune cell (under the assumptions of our model). Immune cells are densely packed in tissue so that these can reliably detect infections. There are approximately $10^{6}$ immune cells per gram of tissue [33] and 1 g of tissue is approximately 1 cm^3^ [34]. Therefore, there is an approximately 100 µm distance between the cells, corresponding to approximately 5 to 10 cell diameters [35]. For the homing radius to be greater than approximately 10 cell diameters, the immune cells need to be slow enough compared with the release rate α of the chemoattractant, namely moving at most α/10 cell diameters per time unit, equation (2.17). In other words, a fast response is detrimental to the accuracy with which the target is reached.

## 4. Conclusion

Statistical fluctuations owing to diffusion set a physical limit for concentration [1] and concentration gradient sensing [5]. In this work, we characterize the change in chemotactic behaviour between a cell in the presence of discrete cue particles rather than a smoothly varying field of chemoattractant. To do this, we construct a simple cell with no internal memory, which we characterize analytically. The choice of a memoryless cell avoids the non-trivial question of search strategy optimization [36] and provides a baseline against which models with varying modes of memory and search strategies can be compared. In the limit of infinite cue particle release rate, corresponding to a smoothly varying chemoattractant field, we derive the deterministic effective velocity and trajectory of the cell. When the cue particle release rate is finite, the cell behaviour becomes stochastic and we derive an effective velocity capturing the mean motion of an ensemble of cells. A characteristic ‘homing radius’ $r_{h}$ emerges, beyond which the cell’s chemotactic index becomes negative (i.e. cells move away from the source on average). This model provides novel insight into how the strength of a chemical source limits the ability of cells to navigate towards it, particularly in the regime where the cell radius is of the order of the persistence length of cell motion. This model could be extended in future to add diffusive dynamics and a finite reorientation time to the cell. Questions of further interest would be the extent to which introducing memory and processing capacity to the cell would increase chemotactic performance, and quantitative comparisons between the predictions of the model and real eukaryotic cells.

## Data Availability

Code which generated the data used in figure 4 is available in this permanent public repository [37]. Supplementary material is available online at [38].
